# Repair of Retinal Degeneration by Human Amniotic Epithelial Stem Cell–Derived Photoreceptor–like Cells

**DOI:** 10.3390/ijms23158722

**Published:** 2022-08-05

**Authors:** Jinying Li, Chen Qiu, Jiayi Zhou, Yang Wei, Weixin Yuan, Jia Liu, Wenyu Cui, Jianan Huang, Cong Qiu, Lihe Guo, Luyang Yu, Zhen Ge

**Affiliations:** 1 MOE Laboratory of Biosystems Homeostasis & Protection, College of Life Sciences, Zhejiang University, Hangzhou 310058, China; 2 College of Life Sciences–iCell Biotechnology Regenerative Biomedicine Laboratory, Joint Research Centre for Engineering Biology, Zhejiang University–University of Edinburgh Institute, Zhejiang University, Haining 314400, China; 3 Key Laboratory of Neuropsychiatric Drug Research of Zhejiang Province, Hangzhou Medical College, Hangzhou 310013, China; 4 Institute of Biochemistry and Cell Biology, Shanghai Institutes for Biological Sciences, Chinese Academy of Sciences, Shanghai 200031, China

**Keywords:** cell therapy, human amniotic epithelial stem cells, retinal degeneration, photoreceptor, immune privilege

## Abstract

The loss of photoreceptors is a major event of retinal degeneration that accounts for most cases of untreatable blindness globally. To date, there are no efficient therapeutic approaches to treat this condition. In the present study, we aimed to investigate whether human amniotic epithelial stem cells (hAESCs) could serve as a novel seed cell source of photoreceptors for therapy. Here, a two–step treatment with combined Wnt, Nodal, and BMP inhibitors, followed by another cocktail of retinoic acid, taurine, and noggin induced photoreceptor–like cell differentiation of hAESCs. The differentiated cells demonstrated the morphology and signature marker expression of native photoreceptor cells and, intriguingly, bore very low levels of major histocompatibility complex (MHC) class II molecules and a high level of non–classical MHC class I molecule HLA–G. Importantly, subretinal transplantation of the hAESCs–derived PR–like cells leads to partial restoration of visual function and retinal structure in Royal College of Surgeon (RCS) rats, the classic preclinical model of retinal degeneration. Together, our results reveal hAESCs as a potential source of functional photoreceptor cells; the hAESCs–derived photoreceptor–like cells could be a promising cell–replacement candidate for therapy of retinal degeneration diseases.

## 1. Introduction

Retinal degeneration accounts for most cases of untreatable blindness globally. Dysfunction and loss of photoreceptors (PRs) are the common endpoints of both inherited and acquired retinal degeneration diseases, such as retinitis pigmentosa and age–related macular degeneration (AMD). As there are currently no effective treatments available for photoreceptor degenerative diseases, there is an urgent need to develop new therapies. Possessing the characteristic of multidirectional differentiation, stem–cell therapy has become a potential therapeutic strategy to replace lost retinal cells and improve vision in correlative patients [1,2,3].

To date, several sources of cells, such as human embryonic stem cells (hESCs) and human induced pluripotent stem cells (hiPSCs), have been explored for their capability to replace damaged or lost retinal cells. Photoreceptor precursors generated from hESCs and hiPSCs were transplanted into mouse models in some preclinical studies, in which they were shown to rescue visual function in vivo [4,5,6]. However, the use of hESCs or hiPSCs faces ethical, immunogenicity, and long–term safety issues that limit their clinical application [7,8,9], although their therapeutic potential has been recorded.

Human amniotic epithelial stem cells (hAESCs), unlike other components of the placenta, are derived from pluripotent epiblasts [10,11,12,13,14]. Our previous study and other reports indicated that hAESCs express some stem cell markers, such as SSEA–3, SSEA–4, SOX–2, and NANOG. Therefore, hAESCs exhibit the capability of multidirectional differentiation [15,16]. In particular, there are several lines of evidence demonstrating that hAESCs express a number of classic markers of neural stem cells and oligodendrocytes, including nestin, β–tubulin III, and myelin basic protein (MBP), and secrete certain neurotrophic factors, suggesting that hAESCs appear to be intrinsically linked to neural cells [17,18,19]. In addition, hAESCs demonstrate immune–privilege because of their high expression of human leukocyte antigen G (HLA–G), as well as low expression of MHC class II molecules HLA–DR and HLA–DQ [20]. Most importantly, hAESCs are genetically stable and show no tumorigenicity upon transplantation into animals and volunteers in the clinic, based on no expression of telomerase hTERT [21]. Pluripotency, low immunogenicity, non–tumorigenicity, and other additional advantages, including easy accessibility and no ethical concerns, make hAESCs attractive for use in the clinical treatment of retinal diseases.

Here, we established an efficient and manipulatable system to induce direct differentiation of hAESCs into PR–like cells by using a set of small molecules. We demonstrate that the retinal structure and visual function of a classic preclinical animal model for retinal degeneration were partially rescued after subretinal transplantation of the hAESCs–PR–like cells. Our study reveals hAESCs as a potential source of functional photoreceptor cells for the treatment of retinal degenerations.

## 2. Results

### 2.1. Identification and Characterization of Human Amniotic Epithelial Stem Cells

**Figure 1:**
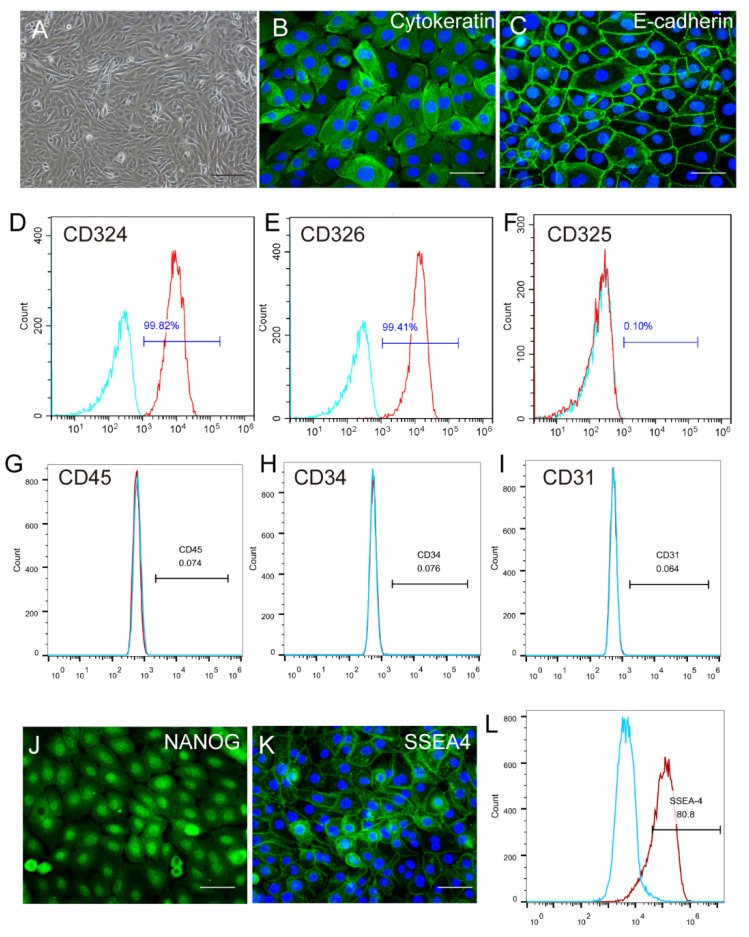
Characteristics of human amniotic epithelial stem cells (hAESCs). (**A**) Phase–contrast microscope image showing isolated hAESCs as a homogeneous population with cobblestone appearance. (**B**–**E**) Nearly all cells showed persistent expression of the representative epithelial marker cytokeratin, E–cadherin (CD324), and E–pcam (CD326) by immunofluorescence and flow cytometry. (**F**) Epithelial–mesenchymal transition marker CD325 was detected using flow cytometry. (**G**–**I**) hAESCs were negative for the hematopoietic lineage markers CD45 and CD34 and endothelial cells markers CD31 as determined using flow cytometry. (**J**–**L**) Strong expression of pluripotency markers NANOG and SSEA4 was determined using immunofluorescence microscopy and flow cytometry. Scale bars, 100 μm in (**A**) and, 50 μm in (**B**,**C**) and (**J**,**K**).

hAESCs were generated from primary amniotic epithelial cell culture. As reported in our previous studies [11,12], hAESCs displayed the typical cobblestone–like appearance of epithelial cells (Figure 1A) and showed high expression of pan–Cytokeratin (Figure 1B), E–cadherin (CD324) (Figure 1C,D), and Epcam (CD326) (Figure 1E), the specific markers of epithelial cells. hAESCs were negative for the epithelial–mesenchymal transition marker N–cadherin (CD325) (Figure 1F), the hematopoietic lineage markers CD45, CD34 (Figure 1G,H), and the endothelial marker CD31 (Figure 1I). Moreover, expression of the pluripotent markers NANOG and SSEA4 supported the plasticity of hAESCs as seed cells (Figure 1J–L).

### 2.2. PR–like Cells Were Obtained from hAESCs by a Two–Step Combined Induction

**Figure 2:**
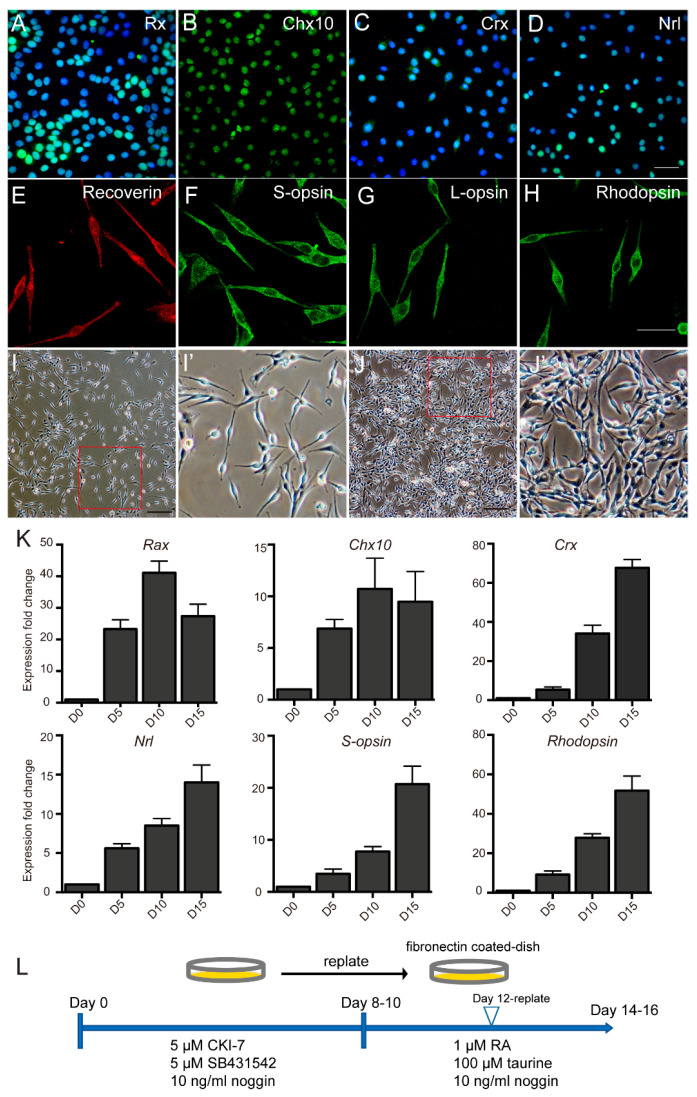
Characteristics of hAESCs–PR–like cells obtained by a two–step combined induction. (**A**–**H**) The expression of signature photoreceptors markers in hAESCs–PR–like cells was demonstrated by immunofluorescence microscopy. Retinal progenitor markers of RX (**A**) and CHX10 (**B**) at day 5, photoreceptor precursor marker of CRX (**C**), early photoreceptor markers of NRL (**D**) at day 10 and mature photoreceptor markers of Recoverin (**E**), S–opsin (**F**), L–opsin (**G**), and Rhodopsin (**H**) at day 14. (**I**,**J**) Morphology of hAESCs–PR–like cells after 2 weeks induction as shown in phase microscope image, with photoreceptor–like shape. (**I′**) and (**J′**) showing the magnification of red frame. (**K**) Expression of photoreceptor markers in induced hAESCs at sequential time points, as measured by quantitative PCR. Expression levels of induced hAESCs were normalized by expression levels of the non–treated hAESCs (D0). (**L**) Schematic of hAESCs differentiation into hAESCs–PR–like cells using a two–step combined induction. Score bars, 50 μm in (**A**–**H**) and 100 μm in (**I**,**J**); error bars represent mean ± SEM.

For photoreceptor induction, we adapted part of the protocol previously reported for hiPSCs and hESCs differentiation [22,23]. Briefly, hAESCs were first treated with a chemical cocktail of 5 μM SB–431542 + 5 μM CKI–7 + 10 ng/mL human noggin (SCN) for about 10 days and then were treated with 100 μM taurine +1 μM retinoic acid +10 ng/mL human noggin (TRN) for about 5 days (Figure S1). In particular, noggin, an inhibitor of the BMP pathway, was added throughout the induction period, which increased the expression level of some photoreceptor precursor markers (Figure S1). By about day 5, strong expression of the signature early retinal progenitor markers RX and CHX10 was detected in differentiated cells by immunostaining (Figure 2A,B). The photoreceptor precursor marker CRX and early photoreceptor marker NRL were detected by about day 10 (Figure 2C,D). Two days later, the differentiated cells were reseeded into 6–well plates precoated by fibronectin. Afterward, immunostaining indicated the strong expression of mature photoreceptor markers of Recoverin, S–opsin, L–opsin, and Rodopsin by about day 14 (Figure 2E–H). Meanwhile, time–course analyses of the key markers’ expression during photoreceptor differentiation further confirmed the induction time length (Figure 2K). Importantly, the morphology of differentiated cells transitioned into a photoreceptor–like shape (Figure 2I,J). According to studies of hESCs and hiPSCs, no fully differentiated PR cells can be achieved in 2D in vitro differentiation, which has been mainly hindered by a lack of functional maturation [24]. Therefore, we defined the differentiated cells above as PR–like cells, although a series of mature photoreceptor markers were detected. In summary, the optimal scheme for inducing hAESCs into PR–like cells was determined, and it is shown in Figure 2L.

### 2.3. The Immune Privilege of hAESCs–Derived PR–Like Cells

**Figure 3:**
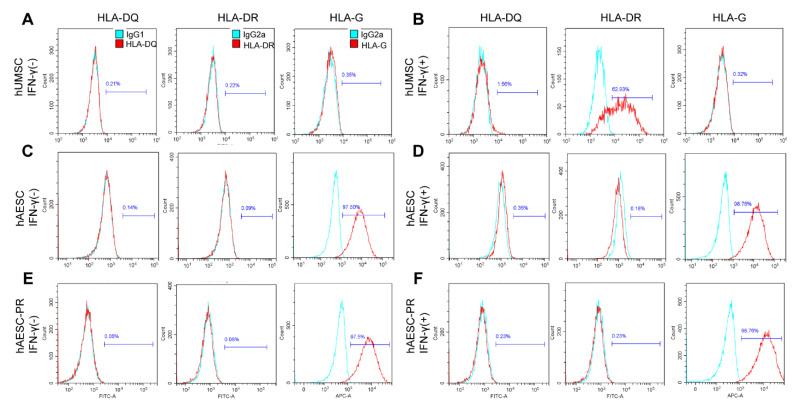
Consistent low immunogenicity in hAESCs and hAESCs−–PR−–like cells under normal and proinflammatory conditions. Flow cytometry analysis of HLA–DQ, HLA–DR, and HLA–G in human umbilical cord mesenchymal stem cells (hUMSC), hAESCs, and hAESCs–PR–like cells in normal culture medium (**A**,**C**,**E**) or with 10 ng/mL IFN–γ treatment for 72 h (**B**,**D**,**F**), indicating consistent low immunogenicity in hAESCs and their derived PR–like cells under normal and proinflammatory conditions. Representative histograms of HLA–DQ, HLA–DR, and HLA–G are shown in red, and the isotype controls are shown in blue.

As a major concern in cell therapy is graft rejection, the immunogenicity of the differentiated cells was detected before subretinal transplantation. HLA–II molecules (–DR, –DQ, and –DP) contribute fundamentally to immune recognition and rejection during allograft, especially HLA–DR and HLA–DQ, because of their high polymorphism. On the other side, the non–classic HLA–G molecule plays a critical role in the maternal immune accommodation and may create an immune–tolerogenic environment to improve the survival of allografts after transplantation by modulating the immune response of regulatory T cells and NK cells [10]. The flow cytometry results demonstrated that significant expression of HLA–DR was detected in IFN–γ–treated human umbilical cord mesenchymal stem cells (hUMSCs), whereas a very low level of HLA–G was detected in hUMSCs (Figure 3A,B). Similar to what was detected in hAESCs (Figure 3C,D), the levels of HLA–DR and HLA–DQ were very low, and the expression of HLA–G was high in hAESCs–PR–like cells, regardless of IFN–γ treatment, which ensures the long–term survival of the differentiated cells within the host retina (Figure 3E,F).

### 2.4. Subretinal Transplantation of hAESCs–PR–like Cells Resulted in Functional Retinal Rescue in RCS Rats

To further determine whether hAESCs–PR–like cells had a therapeutic effect on retinal degeneration in vivo, hAESCs–PR–like cells, differentiated for about 2 weeks in vitro, were transplanted into the subretinal space of RCS rats, the classic preclinical model of retinal degeneration disease. Retinal function and structure were examined 3 weeks after transplantation.

**Figure 4:**
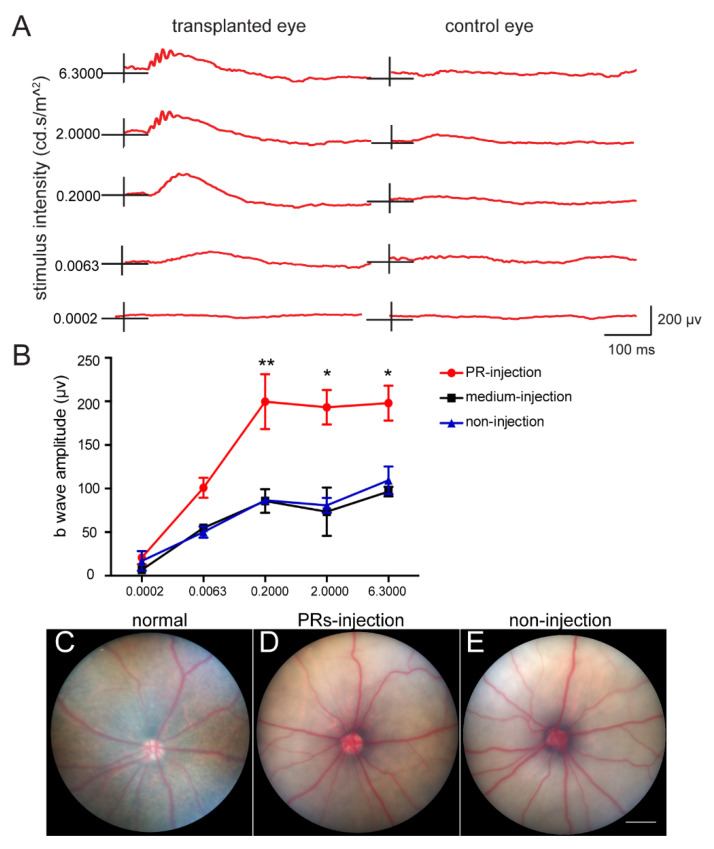
Subretinal transplantation of hAESCs–PR–like cells showed partially functional rescue in RCS rats. (**A**) Representative ERG responses in a transplantation eye and its fellow control eye. (**B**) Mean b–wave amplitudes at different intensity showing a similar preservation pattern compared to groups of control eyes, *n* = 4 per group. (**C**–**E**) Representative color fundus images of normal rat eye, hAESCs–PR–like injection eye of RCS rat, and non–injection eye of RCS rat. 2 mm in (**C**–**E**). Error bars represent mean ± SEM. * *p* < 0.05, ** *p* < 0.01; two–way ANOVA followed by Tukey’s multiple comparisons test.

Functionally, ERG assays revealed significantly improved visual function in cell–grafted eyes when compared with control eyes (Figure 4A). Significantly greater b–wave amplitudes were detected at a series of luminance levels in the dark–adapted state in cell–grafted eyes than in the control groups (Figure 4B). However, color fundus imaging revealed that the transplantation of hAESCs–PR–like cells had no significant effect on fundus pigment restoration (Figure 4C–E). This could be because the transplanted PR–like cells partially restored the PR loss but might not affect the pigmentation defect caused by RPE degeneration.

**Figure 5:**
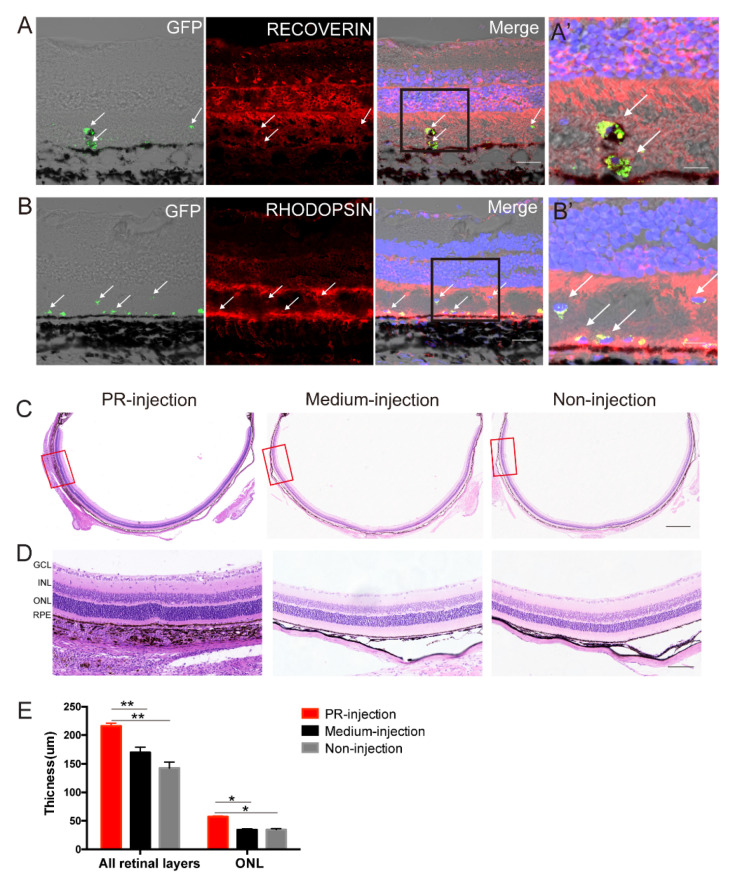
Transplanted hAESCs–PR–like cells provided structural store in RCS rat model. (**A**,**B**) Immunofluorescence staining showed GFP–labeled transplanted hAESCs–PR–like cells coexpressing photoreceptor markers Recoverin (**A**) and Rhodopsin (**B**); higher–magnification images of black frame in (**A′**,**B′**). (**C**–**E**) Representative images of H&E stained retina sections with histological quantifications, showing preservation of ONL and thicker whole retina in subretinal transplantation region compared to groups of control eyes; higher–magnification images of red frame in D, with quantification in E, *n* = 5. Score bars, 50 μm in (**A**,**B**), 20 μm in (**A′**,**B′**), 1 mm in (**C**), and 100 μm in (**D**), error bars represent mean ± SEM. * *p* < 0.05, ** *p* < 0.01; unpaired *t*–test.

Immunostaining analysis demonstrated that the GFP–labelled cells were located in the subretinal space (Figure S2A) and expressed the photoreceptor–specific markers Recoverin and Rhodopsin 3 weeks post–injection (Figure 5A,B). Furthermore, histological analysis demonstrated that organized retinal structure, especially the ONL, was extensively preserved in the region around the cell injection site, which was unlike the obvious ONL loss observed in a similar location in the eyes of rats in medium–injection and non–injection sham groups (Figure 5C,D). These data were corroborated by the quantification of the ONL thickness in the three groups (Figure 5E).

To assess the long–term therapeutic effects of hAESCs–PR–like cells and to substantiate the efficacy and safety of the cell transplantation, cell–grafted and correlated control groups were followed up for 6 weeks after subretinal transplantation. Additionally, immunostaining and histological analysis indicated the survival of the transplanted hAESCs–PR–like cells and their protective effect on retinal degeneration (Figure S2B–F). Noticeably, the potential integration of engrafted hAESCs–PR–like cells into the ONL layer was observed at this time point.

## 3. Discussion

In recent years, the transplantation of healthy photoreceptors to restore visual function in animal models of retinal degeneration has attracted considerable interest. Our study indicated that hAESCs could be induced into photoreceptors–like cells upon a two–step cocktail treatment. hAESCs–derived PR–like cells exhibited the morphology and marker expression of photoreceptors and were found to be capable of partially restoring visual function in a classic preclinical animal model of retinal degeneration. hAESCs, derived from the pluripotent epiblast, show numerous advantages as seed cells for cell therapy, including their plasticity, immunoregulation, absence of tumorigenicity, and lack of ethical concerns [12,13]. Intriguingly, our previous studies and those by others uncovered the neural plasticity potential of hAESC and also revealed the production of neural growth factors by hAESCs [25,26,27]. This can be attributed to the common origin of neural crest cells during embryo development. Moreover, it seems progenitor or unmatured cells may better adapt to the pathological microenvironment in the lesion [28]. Functionally, we have demonstrated the potential therapeutic effect of hAESCs in neural injuries [29,30,31].

A major concern in cell therapy is the graft reaction, which is mainly due to the immunogenicity of transplanted cells. This is a more challenging problem in cell therapy for retinal degeneration since there are no reliable endogenous regeneration strategies for retinal cell replacement, and hESCs– and hiPSCs–derived cells have shown an increased risk of tumorigenicity and rejection. To address the issue in our study, we examined the immunogenicity of hAESCs–derived PR–like cells. Interestingly, the differentiated PR–like cells maintained low levels of HLA–DR and HLA–DQ along with high expression of HLA–G, which were similar to the levels in undifferentiated hAESCs, despite proinflammatory stimulation. This could be attributed to the immune tolerance capacity of hAESCs, based on the high expression of the non–classic MHC class I molecule HLA–G, which smothers natural killer cell recognition and killing, along with the weak expression of MHC class II antigens [11,13,20]. On the other hand, we observed obvious immunogenicity in the IFN–γ treated hUMSCs, which may induce immune responses and graft rejection. This corresponds to the reports indicating that pathological phenotype switch of MSCs and correlated immune disorder after cell transplantation [32,33]. Combined, considering the characteristics of non–tumorigenicity and low immunogenicity and the success of transplantation in the current preclinical study, a decent therapeutic effect of hAESCs–PR–like cells may be expected in clinical use.

With the aim of hAESCs to PR–like cells differentiation, we set up a two–step differentiation protocol with chemical compounds SB–431542/CKI–7/human noggin and retinoic acid/taurine/human noggin, partially adapting the treatment strategy for hiPSCs and hESCs. According to previous studies, the Wnt signaling inhibitor CKI–7 and the Nodal signaling inhibitor SB–431542 are critical small molecule groups that promote the production of retinal progenitors from hESCs or hiPSCs, mimicking the effects of Dkk1 and Lefty–A, respectively [23]. Nevertheless, the BMP pathway inhibitor noggin is a necessary addition in both differentiation steps to induce hAESCs to produce photoreceptors. This is evidenced by the cell morphology switch and photoreceptor marker upregulation. We identified that hAESCs–PR–like cell induction requires two groups of inductors at two stages: the cocktail of SCN for retinal progenitor differentiation and that of TRN for photoreceptor maturation. However, hAESCs derived PR–like cells only exhibited the morphology and some marker expression of photoreceptor and did not form outer segments, suggesting they were not fully differentiated. Our future study will focus on generating more mature photoreceptors derived from hAESCs, by optimizing the differentiation scheme and extending the differentiation time period.

The most intriguing finding in the present study was the rescue of vision function and the retinal structure by hAESCs–derived PR–like cells. These functional effects were closely related to the activities of the transplanted PR–like cells in vivo. After subretinal injection into RCS rats, the majority of the GFP–positive hAESC–PR–like cells did not structurally integrate into the host retina. Thus far, how differentiated PRs function in vivo after transplantation has not been fully ascertained. Some studies indicated that most grafted PR cells did not integrate into recipient tissue but instead remained located in the subretinal space to exchange cytoplasmic intracellular materials with host PRs [34,35,36]. In our study, the partial retinal function improvement 3 weeks after transplantation of hAESCs–PR–like should result from the retinal structure preservation and the endogenous PRs supplemented by material transfer from hAESCs–PR–like cells because all the GFP–labelled cells were observed in the subretinal space. On the other hand, some reports uncovered that a small portion of grafted PRs could integrate into the host ONL depending on the differentiation stage and the degeneration situation of the host retina [28,37]. To this end, it is likely that a few numbers of the implanted hAESC–PR–like cells integrated into the host retina long–term after transplantation. However, the therapeutic effect is not that significant compared to that after short–term transplantation. This could be mainly due to the loss or dysfunction of the transplanted cells, which were rejected as xenografts after a long–term stay without immunosuppressive agents’ administration in the present study. Moreover, the PR progenitors derived from hAESCs with only SCN treatment may adapt the pathological state more efficiently than further differentiated hAESCs–PR–like cells. Future studies will be aimed at comparing cell fate within the retina and the therapeutic effects among hAESCs–derived PR–like cells at different differentiation stages.

Taken together, we may establish an efficient, low–cost, and safety–guaranteed strategy for generating functional PR–like cells from hAESCs. The hAESCs–PR–like cells could be a promising cell–replacement candidate and show therapeutic potential for treating retinal diseases, especially retinal degeneration disease. Further preclinical studies are required to determine the effect of hAESCs–PR–like cells on different types of retinal diseases, considering the disease subtypes in the clinic. To enable clinical treatment, optimal cell doses and delivery schemes, as well as alternative differentiation protocols, should also be evaluated.

## 4. Materials and Methods

### 4.1. Separation of hAESCs

Human amnion membranes were obtained from healthy mothers who provided written informed consent after undergoing a cesarean section, as described in our previous study [22]. The procedure was approved by the Institutional Patients and Ethics Committee of the International Peace Maternity and Child Health Hospital, Shanghai Jiaotong University School of Medicine. All donors were negative for hepatitis A, B, C, and D, as well as HIV–I and Treponema pallidum (TPAB) antibodies. In brief, amniotic membranes were isolated from the placental chorion and washed in HBSS to discard blood cells. The amniotic membrane was incubated with 0.25% trypsin for 20 min at 37 °C in a water bath. Then, hAESCs were centrifuged for 10 min at 300× *g* and counted. hAESCs were cultured in complete culture medium F12/DMEM containing 10% KnockOut Serum Replacement (KSR), 2 mM L–glutamine, 1% nonessential amino acid, 55 μM 2–mercaptoethanol, 1 mM sodium pyruvate, 1% antibiotic–antimycotic (all from Thermo Scientific, Waltham, MA, USA) and 10 ng mL^−1^ human EGF (Peprotech, Rocky Hill, NJ, USA, Cat# AF–100–15) in a humidified atmosphere of 5.5% CO_2_ at 37 °C for 3 to 5 days. The hAESCs used in this research were provided by Shanghai iCELL Biotechnology Co., Ltd. (Shanghai, China) as GMP–grade seed cells.

### 4.2. Differentiation of PR–like Cells from hAESCs

In our study, P0–P3 hAESCs were chosen for investigation. For PR–like cell differentiation, hAESCs were cultured in knockout medium comprising DMEM/F12, 15% (vol/vol) KnockOut serum, 2 mM glutamine, 1 × nonessential amino acids, 1 × antibiotic–antimycotic, 1% B27 supplement (Thermo Scientific), and 1% N2 supplement (Thermo Scientific) in plates pretreated with fibronectin. Then, 5 μM SB–431542 (Sigma–Aldrich, Saint Louise, MO, USA, Cat# C0742), 5 μM CKI–7 (Sigma–Aldrich, Cat# S4317), and 10 ng mL^−1^ human noggin (Peprotech, Cat# 120–10C) were added to the medium for approximately 10 days. Then, 1 μM retionic acid (Sigma–Aldrich, Cat# R2625), 100 μM taurine (Sigma–Aldrich, Cat# T8691), and 10 ng mL^−1^ human noggin (Peprotech) were supplemented during the second week. The medium was replaced every other day, and after 12 days of culture, the cells were passaged to a fibronectin–coated plate at a 1:3 ratio.

### 4.3. Quantitative Reverse–Transcription PCR

Total RNA was isolated from undifferentiated, differentiating hAESCs with an E.N.Z.A. total RNA kit (Omega, BioTek, Norcross, GA, USA) according to the manufacturer’s instructions. Reverse transcription was performed using a ReverTra Ace qPCR RT kit (Toyobo, Osaka, Japan). Quantitative reverse–transcription PCR was performed with the BioRad iCycler real–time PCR detection system (Hercules, CA, USA) with the primers listed in Table S1. To normalize the expression levels, GAPDH was used as an internal control. Quantitative PCR analysis was performed using three biological replicates.

### 4.4. Immunostaining

After fixation with 4% paraformaldehyde in PBS for 15 min, cells were permeabilized using 0.25% Triton X–100 in PBS for 5–10 min and were blocked for 60 min in 5% goat serum. The cells were then incubated for 60 min at room temperature with the following primary antibodies: anti–RAX antibody (Abcam, Cat# ab23340, RRID:AB_447379, 1:200), anti–CHX10 antibody (Sigma–Aldrich, Cat# HPA003436, RRID:AB_1078523, 1:100), anti–CRX antibody (Abnova, Cat# H00001406–M02, RRID:AB_606098, 1:100), anti–Recoverin antibody (Millipore, Burlington, MA, USA, Cat# AB5585, RRID:AB_2253622, 1:200), anti–NRL antibody (Sigma, Cat# SAB1100608, RRID:AB_10605970, 1:200), anti–OPSIN, blue antibody (Millipore, Cat# AB5407, RRID:AB_177457, 1:100), anti–OPSIN, red/green antibody (Millipore, Cat# AB5405, RRID:AB_177456, 1:100), and anti–Rhodopsin antibody (Abcam, Cambridge, UK, Cat# ab5417, RRID:AB_304874, 1:100). Cells were then incubated for 120 min at room temperature with the corresponding secondary antibodies: Alexa Fluor 594–conjugated donkey anti–rabbit IgG (Jackson ImmunoResearch, Philadelphia, PA, USA, Cat# 711–586–152, RRID:AB_2340622), Alexa Fluor 594–conjugated donkey anti–mouse IgG (Jackson ImmunoResearch, Cat# 715–586–150, RRID:AB_2340857), Alexa Fluor 488–conjugated donkey anti–rabbit IgG (Jackson ImmunoResearch, Cat# 711–546–152, RRID:AB_2340619), and Alexa Fluor 488–conjugated donkey anti–mouse IgG (Jackson ImmunoResearch, Cat# 715–545–150, RRID:AB_2340846). Fluorescence images were acquired with a confocal microscope (Zeiss LSM 800, Carl Zeiss Oberkochen, Germany).

### 4.5. Flow Cytometry

To analyze the expression levels of MHC class II antigens (HLA–DR, BioLegend, San Diego, CA, USA, Cat# 307603, RRID:AB_314681 and HLA–DQ, BioLegend, Cat#318104, RRID:AB_604128) and HLA–G (BioLegend, Cat# 335909, RRID:AB_10900805), human umbilical cord mesenchymal stem cells (hUMSCs, Cat# SHTBA0009C1BAC23 obtained from iCell Biological Technology Co., Ltd., Shanghai, China), hAESCs, and hAESCs–PR–like cells were collected after incubation with 10 ng mL^−1^ IFN–γ (Peprotech, East Windsor, NJ, USA, Cat# 300–02–100) for 72 h. The cells were stained with FITC–anti–HLA–DR (isotype control was IgG2a, BioLegend, Cat# 400207, RRID:AB_2884007), FITC–anti–HLA–DQ (isotype control was IgG1, BioLegend, Cat# 400109, RRID:AB_2861401), and APC–anti–HLA–G (isotype control was IgG2a, BioLegend, Cat# 400220, RRID:AB_326468) according to the manufacturer’s instructions, and then they were analyzed by flow cytometry (FACSCalibur; BD Biosciences, Franklin Lakes, NJ, USA). Analyses were performed using three biological replicates.

### 4.6. Animals

RCS rats (3 weeks old at the time of testing, regardless of sex), a known model of retinal disease, were obtained from the Experimental Animal Center of Army Medical University (Chongqing, China) and housed under pathogen–free conditions with a 12 h day–night cycle (lights on at 08:00.). The animals had access to food and water ad libitum, except during test phases. Animals were randomly assigned to the experimental groups (*n* = 5 per group). Data collection and evaluation of all experiments were performed blindly. All procedures involving rats were approved by the Laboratory Animal Care and the Use Committee of Zhejiang University (approval number, ZJU20190038).

### 4.7. Transplantation of hAESCs–PR–Like Cells in RCS Rats

After 14 days of differentiation, hAESCs–PR–like cells were transplanted into 3–week–old RCS rats. In some experiments, hAESCs–PR–like cells were infected with GFP lentivirus for the convenience of detection. In detail, The GFP lentivirus was generated by transfecting HEK–293 cells with a tri–plasmids system of pEGFP–N2, psPAX2, and pMD2G at a ratio of 5:3:2, respectively. The cell medium containing GFP lentivirus was harvested at 48–72 h post–transfection and centrifuged for 10 min at 500× *g*. Then GFP lentivirus solution was concentrated and purified by ultracentrifugation at 50,000–70,000× *g*, 4 °C for 2 h. The undifferentiated hAESCs were infected with GFP lentivirus solution (Hanbio Biotechnology, Shanghai, China) mixed with polybrene (4 μg/mL). After 24 h of infection, the culture medium was changed to differentiation medium for obtaining hAESCs–PR–like cells. The rats were anesthetized by intraperitoneal injection with a mixture of ketamine (Sigma–Aldrich, 70 mg kg^−1^) and xylazine (Sigma–Aldrich, 6 mg kg^−1^). Local anesthetic drops (benoxinate HCl 0.4%; Fischer Pharmaceuticals, Bnei Brak, Israel) were administered. To reduce the efflux of cells, the cornea was punctured with a 30–gauge sterile needle (BD, Biosciences). The conjunctiva was cut at 3 o ‘clock on the temporal side to expose the sclera, and the needle tip was inserted into the sclera at an angle of 15 degrees. Cell suspensions (1.5 × 10^5^ cells in 2 μL of DMEM/F12) were injected into the subretinal space of the left eye through a small scleral incision with a Hamilton needle (34–gauge, Hamilton, Reno, NV, USA). Meanwhile, the right eyes of sham groups were injected with the same dose of medium (DMEM/F12 medium) alone (termed as medium–injection group) or with sham operation of subretinal injection (termed as non–injection group).

### 4.8. Electroretinograms (ERG)

Full–field electroretinograms (ERGs) were recorded after overnight (>12 h) dark adaptation as previously reported [23]. Briefly, the rats were anesthetized in dim red light, and the pupils were dilated with compound tropicamide eye drops. The corneal electrodes were placed on each eye after ophthalmic topical anesthesia, with a subdermal reference electrode and a ground electrode placed in the cheek and tail, respectively. A computerized ERG system (Q450, Roland Consult, Brandenberg an der Havel, Germany) was used to record retinal responses to full–field stimuli. Dim white flashes (−40, −25, −10, 0, and +5 db) under scotopic conditions were used to elicit mixed cone–rod responses (a largely rod–driven response), and the average signal was determined.

### 4.9. Histology and Immunohistochemical (IHC) Staining

The rats were sacrificed at 3 weeks and 6 weeks after implantation. The eyeballs were fixed in 4% formaldehyde for 24 h, dehydrated with 70% alcohol, embedded in paraffin, and serially cut to produce 5 μm thick sections. The slides were stained with hematoxylin and eosin (H&E) according to a standard protocol. For immunostaining, eyecups were directly frozen in OCT (Tissue–Tek, Sakura Finetek, Torrance, CA, USA) and were cut to generate 5 μm–thick sections. Sections were fixed in acetone for 10 min at −20 °C and then were washed with PBS, which was followed by incubation with blocking buffer (1% BSA and 5% HBS in PBS) for 1 h at room temperature. After blocking, sections were incubated for 1 h in a humidified chamber with the following primary antibodies: anti–Recoverin (Millipore, Cat# AB5585, RRID: AB_2253622, 1:200) and anti–Rhodopsin (Abcam, Cat# ab5417, RRID: AB_304874, 1:100). Then, the sections were incubated for 14 h with secondary antibodies. Nuclei were counterstained with DAPI (DAKO, Glostrup, Denmark). Fluorescence images were acquired with a confocal microscope (Zeiss LSM 800, Carl Zeiss).

### 4.10. Statistical Analysis

Statistical analysis was performed using GraphPad Prism 6 (GraphPad, RRID: SCR_002798). Data are presented as the mean ± SEM. Comparisons were performed using unpaired *t*-tests, one–way ANOVA, or two–way ANOVA, followed by Tukey’s multiple comparisons test. The significance level for all tests was set at * *p* < 0.05.

## Data Availability

All data are included in the text and Supplementary Materials.

## Supplementary Materials

The following supporting information can be downloaded at: https://www.mdpi.com/article/10.3390/ijms23158722/s1.
