# Supplementary material for: Repair of Retinal Degeneration by Human Amniotic Epithelial Stem Cell–Derived Photoreceptor–like Cells
Source: Int J Mol Sci. 2022 Aug 5;23(15):8722. doi: 10.3390/ijms23158722 (PMC9369429; doi:10.3390/ijms23158722)
Supplement: Supplementary file 1 [file ijms-23-08722-s001.zip › ijms-1825148-supplementary.pdf]

Supporting Information

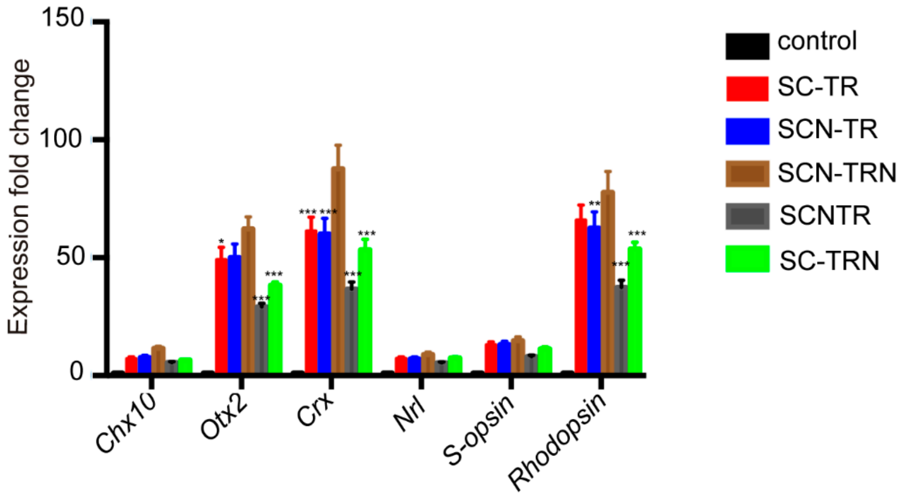

Figure S1. Expression of photoreceptor markers in induced hAESCs treated by different chemical cocktails. Expression of signature photoreceptor markers were determined by quantitative PCR. Expression levels of induced hAESCs were normalized by expression levels of the non-treated hAESCs (control). Retinal progenitor markers: Chx10 and Otx2, photoreceptor precursor marker: Crx, early photoreceptor markers: Nrl, mature photoreceptor markers: S-opsin, and Rhodopsin. S, SB-431542; C, CKI-7; T, taurine; R, retinoic acid; N, noggin. Data are presented as the mean ± SEM of three biological replicates. *p<0.05, **p<0.01 and ***p<0.001 compared with SCN-TRN, two-way ANOVA followed by Bonferroni post-test.

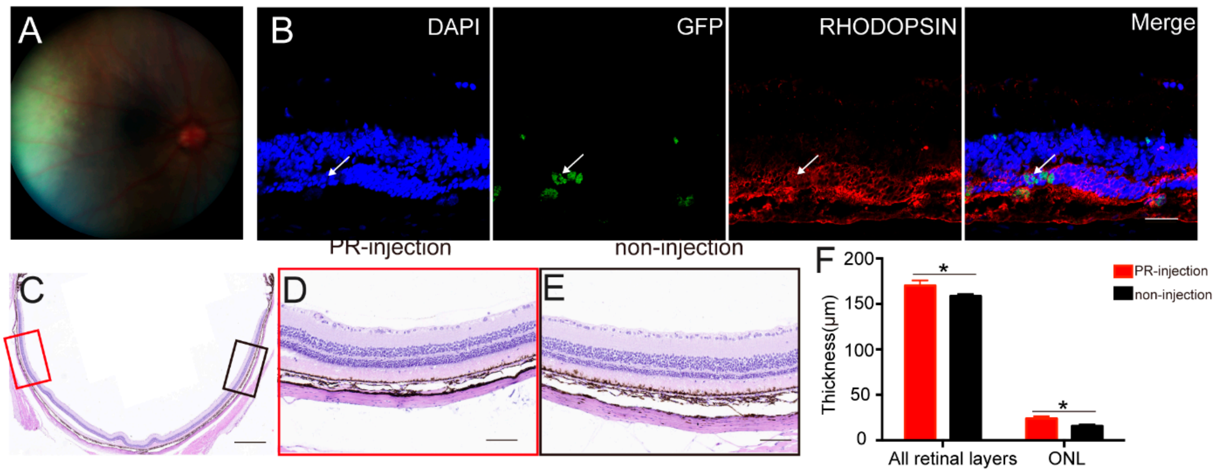

Figure S2. hAESCs-PR-like cells survive and provide long-term (6 weeks) retinal preservation in RCS rats after subretinal transplantation. (A) Fluorescence microscope images of eye cup preparations, showing subretinal GFP+ cells 1 week after transplantation. (B) Immunofluorescence microscopy showed GFP-labeled transplanted hAESCs-PR-like cells coexpressing photoreceptor marker RHODOPSIN. Nuclei were counterstained with DAPI (blue). Note the probable integration of GFP+ hAESCs-PR-like cells within the host ONL; arrows showing transplanted hAESCs-PR-like cells. (C-F) Representative images of H&E stained retina sections with histological quantifications, showing preservation of ONL and thicker whole retina in subretinal transplantation region (indicated by red frame in C and higher-magnification image in D, with quantification in F) as compared with thinner ONL and whole retina in the region distant from graft in the same eye (indicated by black frame in C with higher-magnification image in E, with quantification in F). Scale bars: (B) 50 μm, (C) 500 μm, (D-E) 100 μm. Data are presented as the mean ± SEM. *p<0.05, two-way ANOVA followed by Bonferroni post-test.

Table S1. Primers Used in q-PCR.

Primer	Sequence
Rax-F	CGGCGAAGCGAAACTGTC
Rax-R	CTTAGCCCGTCGGTTCTGG
Chx10-F	CAACGAAGCCCACTACCCA
Chx10-R	CCGTCCTTGGCTGACTTGA
Crx-F	CAGCCTCAGAGTCCCCTTTG
Crx-R	AATAGGAGCTCGGAGACCCA
Nrl-F	CTGGGCTCCACACCTTACAG
Nrl-R	TCCCTGGGTAGTAGCCATGG
S-opsin-F	CCTGGCTACCTGGACCATTG
S-opsin-R	TAGGACTCGCTGCGGTATTTG
Rhodopisn-F	CTTCCTCACGCTCTACGTCAC
Rhodopisn -R	CACGGCTAGGTTGAGCAGG
